# Influence of Fucoidans on Hemostatic System

**DOI:** 10.3390/md11072444

**Published:** 2013-07-12

**Authors:** Nadezhda E. Ustyuzhanina, Natalia A. Ushakova, Ksenia A. Zyuzina, Maria I. Bilan, Anna L. Elizarova, Oksana V. Somonova, Albina V. Madzhuga, Vadim B. Krylov, Marina E. Preobrazhenskaya, Anatolii I. Usov, Mikhail V. Kiselevskiy, Nikolay E. Nifantiev

**Affiliations:** 1 N.D. Zelinsky Institute of Organic Chemistry, Russian Academy of Sciences, Leninsky prospect 47, 119991 Moscow, Russian Federation; E-Mails: ustnad@gmail.com (N.E.U.); bilan@ioc.ac.ru (M.I.B.); vadimkrilov@yandex.ru (V.B.K.); usov@ioc.ac.ru (A.I.U.); 2 V.N. Orekhovich Institute of Biomedical Chemistry, Russian Academy of Medical Sciences, Pogodinskaya str. 10, 119121 Moscow, Russian Federation; E-Mails: natalia.ushakova@ibmc.msk.ru (N.A.U.); marina.preobrajenskaya@ibmc.msk.ru (M.E.P.); 3 Department of Physics, M.V. Lomonosov Moscow State University, Leninskie gory, 119991 Moscow, Russian Federation; E-Mail: kseniazyuzina@ya.ru; 4 N.N. Blokhin Russian Cancer Research Center, Russian Academy of Medical Sciences, Kashirskoe shosse, 24, 115478 Moscow, Russian Federation; E-Mails: anna_el@rambler.ru (A.L.E.); somonova@mail.ru (O.V.S.); takustya@mail.ru (A.V.M.); kisele@inbox.ru (M.V.K.)

**Keywords:** fucoidan, anticoagulant, hemostasis, heparin, thrombin, antithrombin, factor Xa

## Abstract

Three structurally different fucoidans from the brown seaweeds *Saccharina latissima* (SL), *Fucus vesiculosus* (FV), and *Cladosiphon okamuranus* (CO), two chemically modified fucoidans with a higher degree of sulfation (SL-S, CO-S), and a synthetic totally sulfated octasaccharide (OS), related to fucoidans, were assessed on anticoagulant and antithrombotic activities in different *in vitro* experiments. The effects were shown to depend on the structural features of the compounds tested. Native fucoidan SL with a degree of sulfation (DS) of 1.3 was found to be the most active sample, fucoidan FV (DS 0.9) demonstrated moderate activity, while the polysaccharide CO (DS 0.4) was inactive in all performed experiments, even at high concentrations. Additional introduction of sulfate groups into fucoidan SL slightly decreased the anticoagulant effect of SL-S, while sulfation of CO, giving rise to the preparation CO-S, increased the activity dramatically. The high level of anticoagulant activity of polysaccharides SL, SL-S, and CO-S was explained by their ability to form ternary complexes with ATIII-Xa and ATIII-IIa, as well as to bind directly to thrombin. Synthetic per-*O*-sulfated octasaccharide OS showed moderate anticoagulant effect, determined mainly by the interaction of OS with the factor Xa in the presence of ATIII. Comparable tendencies were observed in the antithrombotic properties of the compounds tested.

## 1. Introduction

Thromboembolic disorders, such as venous thromboembolism and arterial thrombosis, are considered to be among the main reasons for cardiovascular diseases. The situation leads to increased utilization of anticoagulant and antithrombotic agents in medical practice. Heparin and its derivatives are usually recommended as support therapy [1,2], but side effects of these medicines, like hemorrhage and heparin-induced thrombocytopenia, force the search for anticoagulants of another nature.

Natural sulfated polysaccharides, such as galactans [3,4,5,6,7], arabinans [8], fucans [3,4,5,9], and fucoidans [3,6,10,11,12,13], present in seaweeds and invertebrates, are regarded as perspective inhibitors of blood coagulation and thrombosis. Influence of these biopolymers on the hemostatic system is determined by the ability of polymeric sulfates to interact with positively charged groups in proteins responsible for hemostasis, leading to the formation of stabilized complexes. The anticoagulant properties of sulfated polysaccharides are mainly connected with thrombin inhibition mediated by antithrombin III (ATIII) and/or heparin cofactor II (HCII), with different efficiencies depending on the structural features of carbohydrates. Other mechanisms, such as direct inhibition of thrombin, are also possible. Thrombin also plays an important role in thrombosis as an inducer of platelet aggregation, and this event may also be controlled by sulfated polysaccharides.

A number of studies revealed certain structure-activity relationships for these macromolecules [3,6,8,11,12,14,15,16]. The main structural features of sulfated polysaccharides, which should be taken into account regarding their anticoagulant and antithrombotic properties, include the monosaccharide composition, the degree and pattern of sulfation, molecular weight, and types of glycosidic bonds. The level of sulfation more than 1.0 was shown to be important for high anticoagulant activity of fucans and galactans [17,18]. High molecular weight fucans demonstrated a greater anticoagulant effect than structurally similar polysaccharides having lower molecular weight [17,19].

The pattern of *O*-sulfation together with the structure of the backbone and branches, but not only total negative charge of sulfates, has a substantial impact on the activity of the biopolymers under discussion (see for example [12]). Thus, in a series of polysaccharides from invertebrates it has been shown that 2-*O*-sulfated (1→3)-linked α-l-galactan, but not an α-l-fucan, with a similar sulfation pattern and molecular size, is a potent thrombin inhibitor mediated by ATIII or HCII [4]. In the case of HCII-mediated inhibition, the major structural requirement for the activity is the presence of selectively 4-*O*-sulfated fucose units [4]. In addition, the linear (1→3)-linked α-l-fucans, enriched in 2,4-di-*O*-sulfated units, were shown to have an amplifying effect on the ATIII-mediated anticoagulant activity [4,9].

Fucoidans from brown algae possess a significantly more complicated structure than fucans from invertebrates due to the presence of numerous branches, non-fucose monosaccharide constituents, and acetates [13]. It was found that, besides ATIII- and HCII-mediated thrombin inhibition activities, which are typical of fucans, the algal fucoidans could also be direct inhibitors of thrombin. For the first time, this behavior was shown for fucoidans from *Fucus vesiculosus* and *Laminaria brasiliensis*, which are built up of alternating (1→3)- and (1→4)-linked α-l-fucose residues sulfated at C-2 and/or C-4, and bearing fucose branches [14]. The similar mechanism of action was shown for fucoidans from the brown seaweeds *Saccharina latissima* (previous name *Laminaria saccharina*) and *Fucus distichus*, but polysaccharides from *Cladosiphon okamuranus* and *Analipus japonicus* were inactive [15]. Notably, a linear arabinan consisting of (1→3)-linked β-l-arabinose residues sulfated at C-2 and/or C-4 was also found to be the potent direct thrombin inhibitor [8].

In this paper we report the results of the study on anticoagulant and antithrombotic activities of three fucoidans from brown seaweeds differed in the monosaccharide composition, types of glycosidic bonds, sulfate content, and sulfation pattern. Chemically *O*-sulfated fucoidan derivatives and structurally related synthetic octasaccharide were studied as well.

## 2. Results and Discussion

### 2.1. Sulfated Carbohydrate Samples

**Figure 1:**
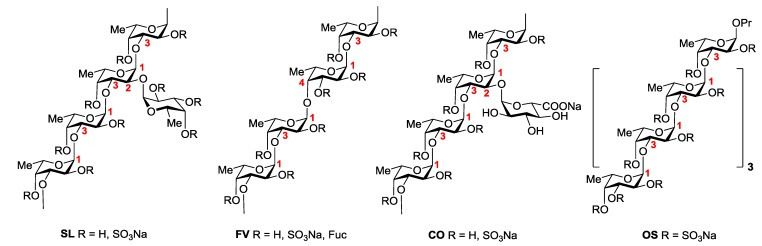
The main structural features of studied samples.

Three samples of native fucoidans were used in this study (see Experimental Section). Their monosaccharide composition and sulfate content are summarized in Table 1. The dominant component of fucoidan SL from *S. latissima* (fraction “F-1.25” in [20]), which demonstrated high anti-inflammatory activity [16], contains poly-(1→3)-α-l-fucopyranosyl backbone sulfated at C-2 and/or at C-4, where approximately each fifth residue bears at C-2 a single sulfated α-l-fucopyranose residue as a branch [20] (Figure 1). The main structural feature of fucoidan FV from *F. vesiculosus* [21] is the backbone consisting of alternating (1→3)- and (1→4)-linked α-l-fucose units, which are sulfated at C-2 and/or C-4. The backbone bears some single α-l-fucose branches, but the degree of branching is lower than that of SL (Figure 1). Fucoidan CO from *C. okamuranus* contains a poly-(1→3)-α-l-fucopyranosyl backbone, sulfated at C-4 and branched at C-2. Its degree of branching is comparable with that of SL, but this fucoidan contains single α-d-glucuronic acid residues as branches instead of fucose residues [22]. It should be noted that fucoidan CO has a relatively low degree of sulfation as compared to the preparations SL and FV (Table 1).

To assess the influence of the degree of sulfation on anticoagulant and antithrombotic activities, chemically *O*-sulfated derivatives of fucoidans SL and CO, namely, samples SL-S and CO-S (Figure 1) were prepared by the reported method [23] (see Experimental Section) and used in this study. According to their enhanced degree of sulfation (Table 1), preparations SL-S and CO-S contain a great number of 2,4-di-*O*-sulfated units. Detailed structural investigation of macromolecules SL-S and CO-S will be published elsewhere.

**Table 1:** Composition * of the samples tested.

Sample	Source	Fuc	Xyl	Man	Gal	Uronic acids	SO_3_Na	Degree of Sulfation **
SL	*S. latissima*	36.7	1.8	0.7	8.4	1.9	39.8	1.3
FV	*F. vesiculosus*	28.5	5.6	2.8	2.9	7.9	26.0	0.9
CO	*C. okamuranus*	42.7	2.0	1.1	1.9	15.1	16.9	0.4
SL-S	Sulfation of SL	26.9	-	1.3	7.1	1.0	46.7	2.0
CO-S	Sulfation of CO	19.7	0.8	0.9	0.7	8.1	45.7	2.4
OS	Synthetic compound	39.4	-	-	-	-	59.1	2.1

* Content (w/w %) of monosaccharides and sulfate (the presence of acetate is not shown); ** Molar ratio SO_3_Na: (Fuc + Gal + UA + Xyl).

In addition to the polymeric samples, synthetic per-*O*-sulfated linear *n*-propyl octa-(1→3)-α-l-fucoside OS (Figure 1) [24], which may be regarded as a linear backbone fragment of polysaccharides SL-S and CO-S, was used as a model to investigate the influence of the molecular weight on the biological activity of fucoidans.

### 2.2. Clotting Assays

General clotting assays were performed with the use of normal plasma, which was incubated with the samples. Commercially available, low-molecular-weight heparin Clexane^®^ (enoxaparin) was chosen as a reference, as this polysaccharide is intensively used in medical practice as a heparinoid anticoagulant with low risk of side effects [2,25,26]. The profiles of anticoagulant activity of the samples and Clexane^®^ were compared.

**Figure 2:**
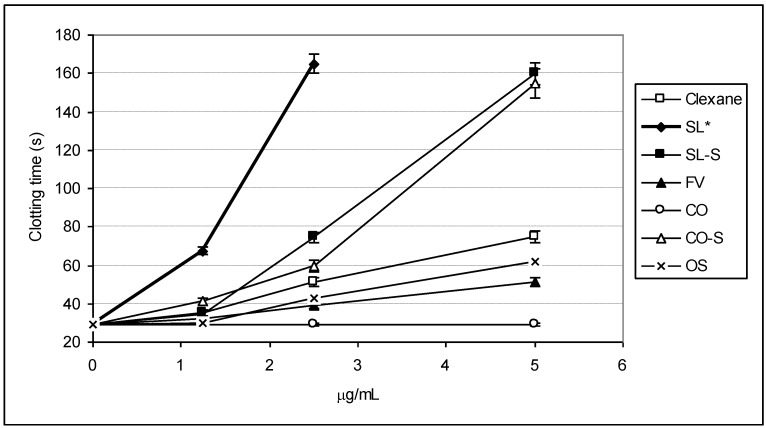
Anticoagulant activity measured by APTT assay.

To evaluate the influence of the fucoidans and their derivatives on the intrinsic pathway of coagulation, the activated partial thromboplastin time (APTT) assays have been performed. The dose-depended changes in the APTT value are shown on Figure 2, and the values of 2APTT (the concentration of a sample, at which double increasing of control value of APTT was observed) are presented in Table 2.

The effect on blood coagulation was shown to depend on the structural features of the tested sample. Among parent fucoidans, the polysaccharide SL demonstrated the highest level of activity, even exceeding that of Clexane^®^. Double increasing of control value of APTT was achieved at a concentration of ~1.1 μg/mL for SL and ~3.3 μg/mL for Clexane^®^. Fucoidan FV showed moderate activity (2APTT ~6.8 μg/mL), while polysaccharide CO was inactive even at a concentration of 100 μg/mL.

* Data for SL at a concentration of 5 μg/mL are omitted, as the APTT value significantly exceeded the upper limit of determination.

**Table 2:** 2APTT and 2TT values for studied compounds.

Sample	2APTT (μg/mL)	2TT (μg/mL)
Clexane^®^	3.32 ± 0.12	2.25 ± 0.03
SL	1.07 ± 0.05	2.43 ± 0.05
SL-S	2.01 ± 0.07	2.20 ± 0.07
FV	6.81 ± 0.03	9.15 ± 0.11
CO	>100	>100
CO-S	2.51 ± 0.09	4.28 ± 0.07
OS	5.10 ± 0.12	16.08 ± 0.23

The degree of sulfation of the polysaccharides was found, in most cases, to influence the activity to a major extent. Per-*O*-sulfation of inactive fucoidan CO gave product CO-S (degree of sulfation 2.4, Table 1) with pronounced anticoagulant properties. This sample prolonged blood coagulation by two times at a concentration of ~2.5 μg/mL. Thus, one of the possible explanations of the low activity of the fucoidan CO can be assigned to the absence of a sufficient amount of sulfate groups in its structure (degree of sulfation 0.4, Table 1).

Interestingly, an opposite situation was observed in the case of the samples SL and SL-S. Additional introduction of sulfates into the structure of SL led to a slight decrease in the anticoagulant effect; however, the sample SL-S (2APTT~2.0 μg/mL) was slightly more active than CO-S. It should be noted that synthetic per-*O*-sulfated octasaccharide OS demonstrated moderate activity (2APTT ~5.0 μg/mL), indicating that its molecular size is not sufficient for achieving a high effect in this test.

To assess the influence of the studied compounds on the extrinsic pathway of coagulation, the value of Prothrombin time (PT) was measured. All samples, except CO, slightly increased PT in a dose-dependent manner (0.5–2 s, data not shown) within the concentration range used in the APTT test. According to literature data [15], significant changes in PT could be expected when fucoidan is applied at concentrations higher than 10 μg/mL.

**Figure 3:**
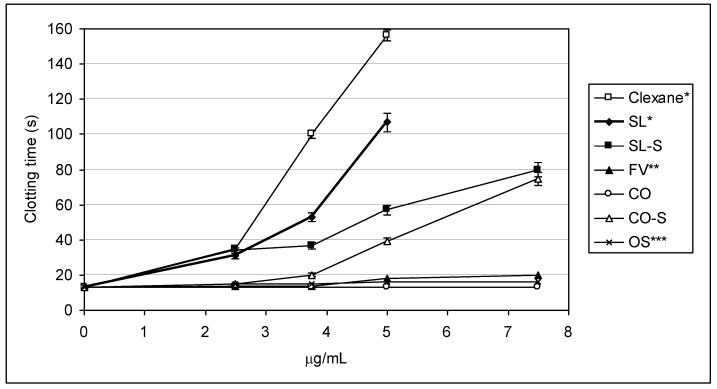
Anticoagulant activity measured by TT assay.

The influence of the samples on thrombin-induced clot formation was also investigated. The value of Thrombin time (TT) was measured at several concentrations (see Figure 3) to again show different profiles of activity for the tested samples. The values of 2TT (the concentration of a sample, at which double increasing of control value of TT was observed) were also calculated (Table 2). The determined 2TT-values for Clexane^®^ (~2.2 μg/mL), SL (~2.4 μg/mL), and SL-S (~2.2 μg/mL) suggested that these preparations possess comparable effects. Increasing polysaccharide concentration gave quite different responses. Thus, at a concentration of ~3.8 μg/mL Clexane^®^ prolonged blood coagulation by eight times, while SL and SL-S gave prolongation only by four and three times, respectively. It is noticeable that, similarly to the results of the APTT test, the chemically sulfated polysaccharide SL-S exhibited slightly lower activity than parent fucoidan SL.

* Data for Clexane^®^ and SL at a concentration of 7.5 μg/mL are omitted, as the TT value significantly exceeds the upper limit of determination; ** FV in a concentration of 15 μg/mL showed TT = 52 s; *** OS in a concentration of 21 μg/mL showed TT = 60 s.

The fucoidan CO was inactive in the TT assay even at a concentration of 100 μg/mL, while its sulfated derivative CO-S at a concentration of ~7.5 μg/mL demonstrated a comparable effect with preparation SL-S at the same concentration. Significant activity of the samples SL, SL-S, and CO-S in this test could be explained by their ability to interact with thrombin. Similarly to the results of the APTT test, fucoidan FV showed moderate inhibitory effect in TT assay (the 2TT value was ~9.2 μg/mL). This can be due to its slightly lower degree of sulfation (0.9, Table 1) in comparison with SL, or to different presentations of sulfates because of another type of the backbone (Figure 1). Although the octasaccharide OS was structurally related to the backbones of SL-S and CO-S, its activity was lower (2TT ~16.1 μg/mL). This indicates again that longer and probably branched molecules are required for high anticoagulant effect.

### 2.3. Effect of the Compounds on the Inactivation of Thrombin and Factor Xa in the Presence and in the Absence of Antithrombin III

**Figure 4:**
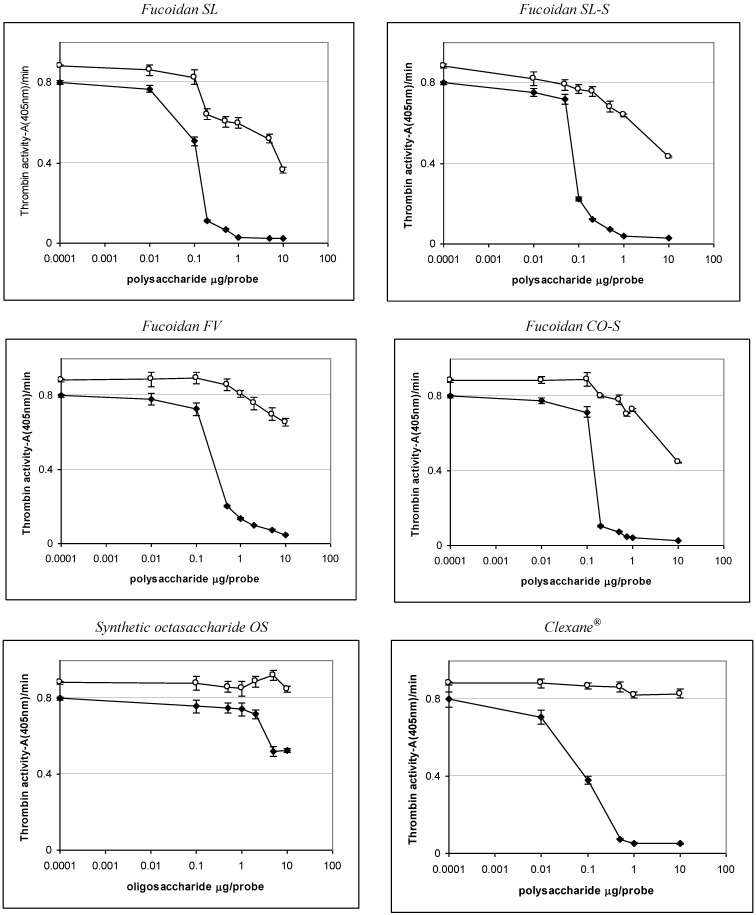
Effect of the samples on thrombin inactivation in the presence and in the absence of ATIII. (♦) experiments with ATIII, (○) experiments without ATIII.

**Figure 5:**
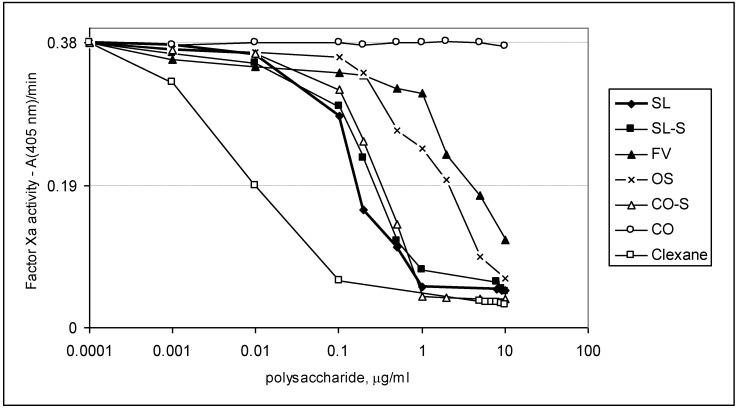
Effect of the samples on factor Xa inactivation in the presence of ATIII.

To investigate further the mechanism of anticoagulant action of fucoidans and their derivatives, the experiments with purified proteins were performed. These studies were based on the assay of amidolytic activity of thrombin (IIa) or factor Xa using chromogenic substrates, as described previously [8,14,15]. The ability of samples to inhibit thrombin and factor Xa was assessed in the presence and in the absence of ATIII. In addition to Clexane^®^, another heparin-related drug, Arixtra^®^ (fondaparinux, based on synthetic pentasaccharide), was used as a reference. The results are shown in Table 3 and Figure 4, Figure 5.

**Table 3:** Inhibition of thrombin and factor Xa.

Sample	IC_50_ (μg/mL)		
	АТIII + thrombin	+thrombin	АТIII + Xa
SL	0.76 ± 0.04	45.86 ± 0.58	1.06 ± 0.04
SL-S	0.47 ± 0.02	55.86 ± 1.07	1.94 ± 0.08
FV	2.1 ± 0.08	No *	28.22 ± 0.95
CO	no	no	No
CO-S	0.88± 0.03	58.81 ± 1.12	2.06 ± 0.09
OS	No **	no	12.94 ± 0.98
Clexane^®^	0.59 ± 0.02	no	0.059 ± 0.002
Arixtra^®^	no	no	0.0065 ± 0.0003

* Not observed, but only 25% inhibition was observed at a concentration of 59.0 μg/mL; ** Not observed, but only 34% inhibition was observed at a concentration of 59.0 μg/mL.

The preparations SL, SL-S, CO-S, and FV were shown to interact with thrombin both in the presence and in the absence of ATIII, while heparinoid Clexane^®^ was active only in the presence of ATIII. Effectiveness of binding to thrombin in the presence of ATIII was high for the samples SL, SL-S, CO-S, and Clexane^®^ (IC_50_ < 1.0 μg/mL), but it was slightly lower for the fucoidan FV (IC_50_ ~2.1 μg/mL). The fucoidan CO was inactive in all tested concentrations.

The synthetic octasaccharide OS demonstrated more than 100 times lower anti-IIa activity in the presence of ATIII than the polysaccharides SL, SL-S, and CO-S, with the same backbone. This result could be compared with known data for heparinoids. Thus, synthetic pentasaccharide Arixtra^®^ did not inhibit thrombin activity in the presence of ATIII even at a concentration of 100 μg/mL, while the polysaccharide Clexane^®^ demonstrated significant inhibitory effect in this experiment (IC_50_ < 1.0 μg/mL). It was established earlier [27,28,29] that more than 16 monosaccharide units in the chain of the heparinoid structure are required for the formation of a ternary complex with thrombin and ATIII.

For direct thrombin inhibition, higher concentrations of polysaccharides were required. Thus, the samples SL, SL-S, and CO-S showed 50% inhibition at concentrations of ~45.9, ~55.9, and ~58.9 μg/mL, respectively, while the fucoidan FV demonstrated only 25% inhibition at a concentration of 59.0 μg/mL.

The fucoidan CO, as well as the synthetic octa-(1→3)-α-l-fucoside OS, were found to be unable to interact directly with thrombin. It is interesting to mention that, according to the theoretical prediction, the high activity of linear sulfated (1→3)-β-l-arabinan was explained by the specific structure of its octasaccharide fragment [8]. The different biological properties of these two octasaccharides demonstrate, once more, the importance of the carbohydrate structure for the biological activity of sulfated polysaccharides.

The polysaccharides SL, SL-S, and CO-S efficiently bind to factor Xa only in the presence of ATIII (the values of IC_50_ were ~1.1, ~1.9, and ~2.0 μg/mL, respectively), but their activity was significantly lower than that for heparinoids Clexane^®^ and Arixtra^®^ (the values of IC_50_ were ~0.059 and ~0.0065 μg/mL, respectively). The fucoidan FV showed 50% inhibition only at a concentration of ~28.0 μg/mL. Surprisingly, the synthetic per-*O*-sulfated octasaccharide OS demonstrated moderate anti-Xa activity in the presence of ATIII (IC_50_ were ~13.0 μg/mL), even exceeding the effect of polysaccharide FV. Neither fucoidans, nor heparinoids, bind to factor Xa in the absence of ATIII (data not shown).

The tendencies found in amidolytic experiments (see Table 3 and Figure 4, Figure 5, showing anti-IIa and anti-Xa activities) for the studied samples correlated well with the results obtained in clotting assays. Thus, the polysaccharides SL, SL-S, and CO-S, enriched in 2,4-di-*O*-sulfated (1→3)-linked α-l-fucose units demonstrated high effects in clotting assays and also possessed significant anti-IIa and anti-Xa activities. These results are in good agreement with the data obtained previously for linear highly sulfated fucans from invertebrates [4,9,14]. Moreover, it is noticeable that inhibition of thrombin by these polysaccharides was performed both in the presence and in the absence of ATIII, which coincides well with the published data for other branched fucoidans from brown seaweeds [14,15].

### 2.4. Influence on Platelets Aggregation

**Figure 6:**
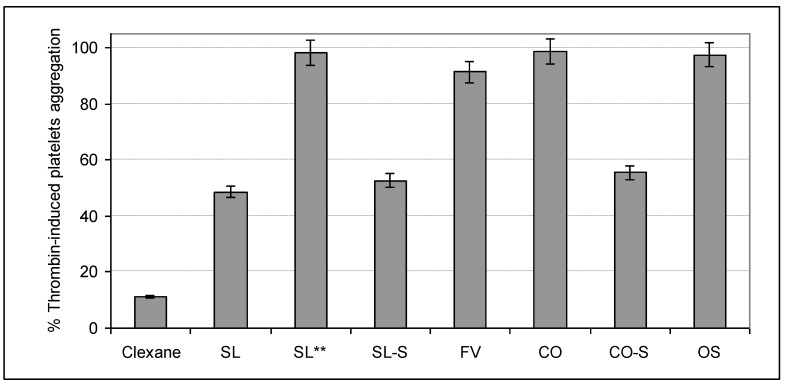
Influence of the samples on thrombin-induced platelets aggregation *.

**Figure 7:**
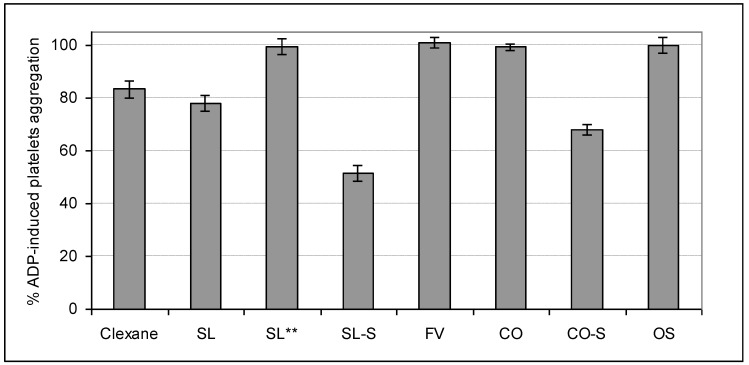
Influence of the samples on ADP-induced platelets aggregation *.

The ability of the studied samples to influence on the cell-regulated hemostasis was assessed in experiments with platelets rich plasma (PRP). Two types of inducers of platelets aggregation, namely thrombin and adenosine diphosphate (ADP), were used. All samples were tested at a concentration of 100 μg/mL, while the fucoidan SL was also studied at a concentration of 10 μg/mL (Figure 6, Figure 7).

* Activities of studied samples (100 μg/mL) were tested in the presence of 0.5 U of thrombin; ** Activity of fucoidan SL at a concentration of 10 μg/mL.

* Activities of studied samples (100 μg/mL) were tested in the presence of 10 μm of ADP; **Activity of fucoidan SL at a concentration of 10 μg/mL.

The polysaccharides SL, CO-S, and SL-S, as well as heparinoid Clexane^®^, efficiently inhibited thrombin-induced platelets aggregation. The 51% inhibition was observed for SL, 48% for SL-S, 42% for CO-S, and 88% for Clexan^®^, while the fucoidans FV and CO, the synthetic octasaccharide OS, as well as SL (at a concentration of 10 μg/mL), were inactive in this test. Thrombin is considered as the main protein target in this assay and, thus, the results obtained demonstrate comparable tendencies described above for the amidolytic assay.

The preparations SL, SL-S, and CO-S demonstrated a statistically significant effect on ADP-induced platelets aggregation exceeding that for Clexane^®^. Contrary to the mechanism of thrombin-induced platelets aggregation, the polysaccharide inhibitors interact directly with special receptors on the surface of platelets but not with the inducer of aggregation. That is why the observed values of inhibitory effects of the tested samples differed from those measured in the previous experiment. Inhibition of aggregate formation in PRP was 50% for SL-S, 32% for CO-S, and 22% for SL. The fucoidans FV and CO, the synthetic octasaccharide OS, as well as SL (at a concentration of 10 μg/mL) were inactive again.

## 3. Experimental Section

### 3.1. Preparation of the Samples

Preparation of the fucoidan SL (“fraction F-1.25” in [20]) from the brown seaweed *S. latissima* was described earlier [20]. A crude fucoidan preparation extracted from *F. vesiculosus* was purified by decoloration with NaClO_2_ in dilute HCl [30] followed by precipitation with cetyltrimethylammonium bromide and transformation into sodium salt [31], giving rise to purified fucoidan FV. The polysaccharide CO was received as a gift from Dr. M. Iho (South Product Co., Suzaki, Japan).

The fucoidans SL and CO were subjected to *O*-sulfation under acid-promoted conditions [23], transformed into sodium salts and desalted by column chromatography on Sephadex G-15 (2.5 × 70 cm, elution with water) to obtain the corresponding oversulfated polysaccharides SL-S and CO-S, respectively. The octasaccharide OS was synthesized from l-fucose [24] and characterized by mass spectrometry (ESI-MS), as well as by ^1^H and ^13^C NMR spectroscopy.

The monosaccharide composition and sulfate content of the samples were determined as described earlier [32] and summarized in Table 1.

### 3.2. General Coagulation Assays

APTT and PT were determined with an SRA-R Evolution^®^ hemostasis analyzer (Diagnostica Stago, USA) according to the established procedure and using standard reagents (Diagnostica Stago). A solution (15 μL) containing 15 μg, 7.5 μg, or 3.75 μg of a sulfated carbohydrate sample in saline solution (0.9% NaCl) was added to the normal platelet-depleted citrated plasma (3 mL). Low-molecular-weight heparin Clexane^®^ (manufactured by Sanofi) was used for the comparison of anticoagulant activity of the samples. The saline solution was used as a control. The samples were incubated at 37 °C for 1 min.

A kit, “Thrombin test” (Renam, Russia), was used for determination of thrombin time. A saline solution with a sulfated carbohydrate sample (20 μL) was added to the normal plasma (80 μL). The mixture was incubated at 37 °C for 1 min, and a saline solution (100 μL) of stabilized thrombin (10 U/mL) was added. The saline solution was used as a control. The time of clot formation was then determined.

### 3.3. Amidolytic Assays

Determination of amidolytic activity of thrombin was performed using “ReaChrom ATIII test” kit (Renam, Russia) as described previously [15]. A solution of ATIII (0.2 U/mL, 50 μL) or the buffer (50 μL) was added to a solution of a sulfated carbohydrate sample (0.001–10 μg) in buffer (0.15 μM Tris-HCl, pH 8.4, 20 μL), followed by the addition of an aqueous solution (50 μL) of human thrombin (5.0 U/mL). The mixture was incubated at 37 °C for 3 min, and 50 μL of synthetic chromogenic substrate was added. After 2 min the reaction was quenched by 220 μL of 50% acetic acid. The absorbance of *para*-nitroaniline was measured at 405 nm on an Ultospec II spectrophotometer (LKB, Switzerland).

Determination of amidolytic activity of factor Xa was performed using “ReaChrom Heparin” (Renam, Russia) kit. A solution of ATIII (0.5 U/mL, 50 μL) or the buffer (50 μL) was added to a solution of a sulfated carbohydrate sample (0.0001–10 μg) in the buffer (0.15 μM Tris-HCl, pH 8.4, 20 μL). Then an aqueous solution of the factor Xa (2.0 U/mL, 50 μL) was added. The mixture was worked-up, treated with chromogenic substrate, and analyzed as described above.

### 3.4. Inhibition of Platelets Aggregation

Blood with citrate buffer (9:1) was centrifuged at 1000 rpm for 5 min, and platelets rich plasma was collected from different tubes and combined. A saline solution (10 μL) containing 45 μg of a sulfated carbohydrate sample was added to 450 μL of this plasma. The saline solution was used as a control. The samples were incubated at 37 °C for 2 min, then 10 μm of ADP or 0.5 U of thrombin was added, and light transmittance was measured on a Chrono-Log aggregometer. The data were transformed to percent of platelets aggregation using the established software. The control was considered as 100% of platelets aggregation.

### 3.5. Statistical Analysis

All experiments were performed in quadruplicate (*n* = 4). The results are presented as mean ± S.D. Statistical significance was determined with Student’s *t* test. The *P* values less than 0.05 were considered as significant.

## 4. Conclusions

The effect of the fucoidans on blood coagulation and platelets aggregation was shown to depend on their structural features. Thus, the branched polysaccharides SL, SL-S, and CO-S, enriched in 2,4-di-*O*-sulfated (1→3)-linked α-l-fucose units demonstrated high effect in clotting assays, while the fucoidan FV built up of alternating (1→3)- and (1→4)-linked α-l-fucose residues with a degree of sulfation of 0.9 was remarkably less active. The polysaccharide CO with the (1→3)-linked α-l-fucose backbone containing α-d-glucuronic acid branches at C-2 with the degree of sulfation 0.4 did not influence on blood coagulation, and this feature could be explained by the absence of a sufficient amount of sulfate groups in its structure, or by the presence of glucuronic acid side residues. The tendencies found in clotting assays correlated with the results obtained in experiments with individual proteins. High anti-IIa and anti-Xa activities were shown for the samples SL, SL-S, and CO-S in the presence of ATIII. The fucoidan FV demonstrated moderate effects under these conditions, while the fucoidan CO was inactive. It is noticeable that the branched polysaccharides with the degree of sulfation ≥0.9 (SL, SL-S, CO-S, and FV) were also shown to be direct thrombin inhibitors and, hence, they differ from heparinoids and linear fucans from invertebrates. The synthetic per-*O*-sulfated octasaccharide OS, which was structurally related to the polysaccharides SL, SL-S, and CO-S, possessed a moderate effect on clot formation connected mainly with its moderate anti-Xa activity. This result indicated that longer, and probably branched, fucoidan fragments are required for the efficient inhibition of blood coagulation. The most active samples SL, SL-S, and CO-S also exhibited a statistically significant inhibitory effect on platelets aggregation mediated by ADP and thrombin.
